# Efficacy of direct-acting antiviral regimens for acute HCV infection: a systematic review and meta-analysis

**DOI:** 10.1186/s12985-026-03307-x

**Published:** 2026-10-07

**Authors:** Nada Mansour, Ramy M. F. Elbarody, Ahmed M. Kamel

**Affiliations:** 1 Clinical Pharmacy Department, Faculty of Pharmacy, Cairo University, Cairo, Egypt; 2 Department of Gastroenterology, King Salman Specialist Hospital, Hail Health Cluster, Hail, Saudi Arabia

**Keywords:** Hepatitis C, Acute hepatitis C, Recently acquired HCV, Direct-acting antivirals, Glecaprevir/pibrentasvir, Sustained virological response, Treatment duration, Meta-analysis

## Abstract

**Background:**

Acute and recently acquired hepatitis C virus (HCV) infection clusters in men who have sex with men living with HIV and in people who inject drugs, where early cure benefits both the individual and transmission control. The best interferon-free dual direct-acting antiviral (DAA) regimen and treatment duration, including short courses, remain undefined.

**Methods:**

MEDLINE, Scopus, and the Cochrane Central Register of Controlled Trials were searched to 1 July 2026 for studies of interferon-free dual-DAA therapy in acute or recently acquired HCV reporting SVR12. Each study was appraised as a single-arm case series, and proportions were pooled by random-intercept logistic regression. Pre-specified subgroups were regimen, treatment duration, study design, and HIV status.

**Results:**

Fourteen studies (19 single-arm analysis units; 1078 treated patients) were included. Pooled SVR12 was 97.7% (95% CI 94.7–99.1; 1043/1078; I² = 30.4%). SVR12 rates increased with treatment duration, from 84.8% (74.1–91.6) at 4 weeks and 91.7% (86.2–95.1) at 6 weeks to 99.4% (97.5–99.9) at 8 weeks and 97.4% (91.7–99.2) at 12 weeks (between-subgroup *p* < 0.001), whereas it did not differ by regimen (*p* = 0.608), study design (*p* = 0.764), or HIV status (*p* = 0.975). The pooled estimate was stable across sensitivity analyses (trials only, exclusion of the high-risk study, and leave-one-out).

**Conclusion:**

Dual-DAA therapy was associated with a high pooled SVR12 in acute and recently acquired HCV, with cure differing by treatment duration. These findings support early treatment and favour a course of at least eight weeks. Given the predominantly single-arm evidence and the partial confounding of regimen with duration, randomised duration-comparison trials are needed to confirm the optimal short-course strategy.

**Graphical abstract:**

**Figure:**
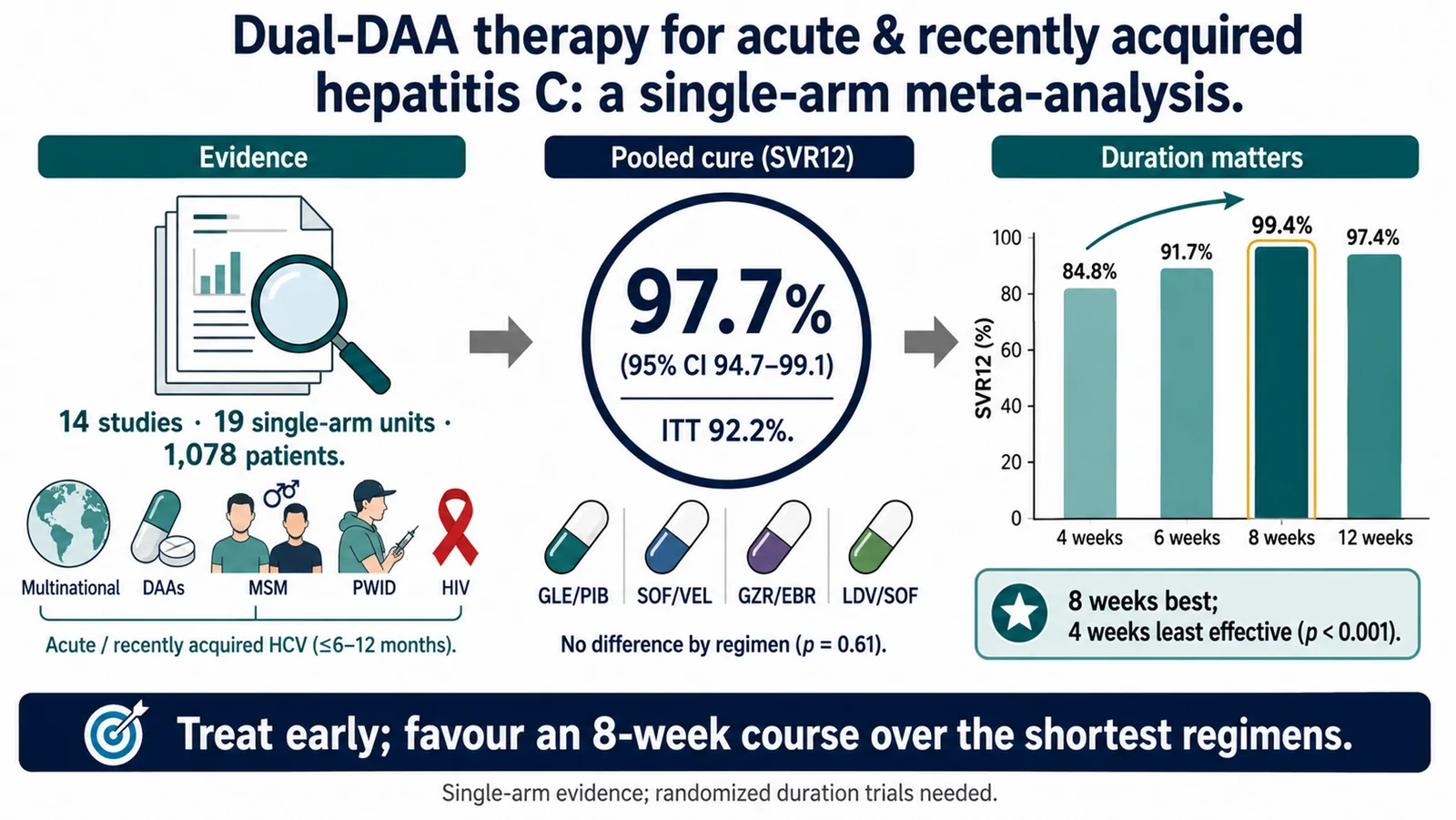

**Supplementary Information:**

The online version contains supplementary material available at https://doi.org/10.1186/s12985-026-03307-x.

## Introduction

Hepatitis C virus (HCV) infection remains a major global health problem, with an estimated 50 million people living with chronic infection and several hundred thousand deaths each year from cirrhosis and hepatocellular carcinoma [1]. The World Health Organization has set targets to eliminate HCV as a public-health threat by 2030, which require both scaled-up testing and rapid, effective treatment [2]. The introduction of interferon-free, direct-acting antiviral (DAA) therapy transformed the management of chronic HCV, producing sustained virological response (SVR) rates above 95% with short, well-tolerated, pangenotypic regimens [3–5].

Alongside chronic infection, acute and recently acquired HCV has become concentrated in identifiable key populations, particularly men who have sex with men living with HIV and people who inject drugs, among whom ongoing transmission sustains new infections [6–8]. Acute or recently acquired HCV is generally defined, following European AIDS Treatment Network criteria, as infection of an estimated duration of six to twelve months or less, documented by recent seroconversion or new viraemia after a previously negative test [9]. Approximately one quarter of individuals clear the virus spontaneously, most within the first six months, while the remainder progress to chronic infection [10, 11].

Early treatment benefits the individual and reduces onward transmission, supporting micro-elimination in high-incidence networks, and has been associated with falling incidence where DAA access is unrestricted; current guidelines increasingly endorse treating recently acquired infection rather than awaiting spontaneous clearance [4, 12–14]. Because early infection carries lower viral diversity, several groups have tested shortened courses, reporting high cure after eight weeks but lower and variable response after four to six weeks, and the value of reliable cure is heightened by the substantial risk of reinfection in these populations [15–19].

A prior meta-analysis examined immediate treatment of recent HCV but was restricted largely to standard-duration therapy and preceded much of the short-course glecaprevir/pibrentasvir programme [20], while the most relevant Cochrane review addressed chronic infection [21]. We therefore performed a systematic review and single-arm proportion meta-analysis to estimate the pooled SVR12 achieved with interferon-free dual-DAA regimens in acute or recently acquired HCV, overall and stratified by regimen, treatment duration, study design, and HIV status.

## Methods

### Protocol, registration, and reporting

This systematic review with single-arm proportion meta-analysis was conducted and reported in accordance with the Preferred Reporting Items for Systematic Reviews and Meta-Analyses (PRISMA) 2020 statement [22], and the Meta-analysis Of Observational Studies in Epidemiology (MOOSE) recommendations were followed for the observational component [23]. The protocol was registered prospectively with the International Prospective Register of Systematic Reviews (PROSPERO; registration CRD420261464486) before data extraction, and the completed PRISMA 2020 checklist is provided in the Supplementary material. The modified intention-to-treat or per-protocol (mITT/PP) proportion was adopted as the primary effect measure, with the intention-to-treat (ITT) proportion retained as a pre-specified sensitivity analysis; and pooling was performed with a random-intercept logistic regression model in preference to the Freeman–Tukey double-arcsine transformation [24].

### Eligibility criteria

Eligible studies enrolled adolescents and adults (≥ 12 years) with acute or recently acquired HCV infection, defined per the European AIDS Treatment Network or European Association for the Study of the Liver consensus as an estimated infection duration of six to twelve months or less, in either HCV monoinfection or HIV coinfection and in any risk group [9]. The intervention was any interferon-free, oral, dual DAA regimen of any treatment duration. Randomised controlled trials, non-randomised single-arm interventional trials, and prospective or retrospective cohorts and case series were eligible. Studies of interferon-containing regimens, single-DAA monotherapy, triple-DAA regimens, or DAA-plus-ribavirin combinations in which the dual-DAA effect could not be isolated were excluded, as were studies not reporting SVR12 or data permitting its calculation. The primary outcome was SVR12.

### Information sources and search strategy

MEDLINE (via PubMed), Scopus, and the Cochrane Central Register of Controlled Trials were searched from 1 January 2011 to 1 July 2026 without language restriction, and ClinicalTrials.gov and the World Health Organization International Clinical Trials Registry Platform were searched for completed but unpublished trials. Search terms combined three concept blocks (1) acute or recently acquired HCV, (2) direct-acting antiviral agents, and (3) hepatitis C. Searches were supplemented by backward and forward citation tracking of included studies and relevant prior reviews and by screening of conference proceedings. Full line-by-line strategies for each database are provided in the Supplementary material.

### Study selection and data extraction

Records were de-duplicated, after which two reviewers independently screened titles and abstracts and then full texts against the eligibility criteria, with disagreements resolved by discussion or by a third reviewer. Two reviewers then independently extracted the citation, country, design, registration identifier, risk group, HIV status, HCV genotype, the acute-HCV definition applied, regimen, treatment duration, sample size, and the SVR12 numerator and denominator under both ITT and mITT/PP definitions. Studies that randomised treatment duration or reported more than one regimen were decomposed into mutually exclusive single-arm analysis units, so that each participant contributed to exactly one unit.

Because no eligible study provided a randomised dual-DAA-versus-comparator contrast for SVR12, every included study was decomposed into single-arm SVR12 proportions and appraised as a case series, irrespective of its originating design. The single randomised trial, REACT, randomised treatment duration rather than regimen, so each duration arm was handled as an independent single-arm proportion; the multi-regimen cohorts, Chromy and Martin-Carbonero, were partitioned by regimen and duration into mutually exclusive patient sets.

### Risk-of-bias assessment

The methodological quality of every included study was appraised as a single-arm case series using the Joanna Briggs Institute Critical Appraisal Checklist for Case Series [25, 26], because each study, irrespective of its original design, contributed a within-arm SVR12 proportion rather than a between-group comparison. Nine checklist items were each rated as yes, no, unclear, or not applicable, and an overall rating of low, moderate, or high risk of bias was assigned on the basis of the number of items satisfied, with mandatory items requiring complete participant inclusion and a fully reconcilable SVR12 numerator and denominator for a low-risk classification. Each study was assessed independently by two reviewers, with disagreements resolved by discussion or a third reviewer. For the single randomised controlled trial, the Cochrane Risk of Bias 2 tool was additionally applied and is reported in the Supplementary material.

### Statistical analysis

SVR12 proportions were pooled using a random-effects meta-analysis of single proportions fitted as a random-intercept logistic regression (generalised linear mixed) model on the logit scale, with between-study heterogeneity (τ²) estimated by maximum likelihood [27, 28]. Study-specific proportions with exact Clopper–Pearson confidence intervals and the back-transformed pooled estimate with its 95% confidence interval are reported as percentages, and a 95% prediction interval was derived from a t-distribution. This model was preferred to the Freeman–Tukey double-arcsine transformation because the latter can yield misleading pooled estimates for proportions near one [24]. Heterogeneity was quantified with the I² statistic, τ², and the Q test. The mITT/PP proportion was the primary effect measure and the ITT proportion a pre-specified sensitivity analysis. Pre-specified subgroup analyses examined regimen, treatment duration (4, 6, 8, and 12 weeks), study design, and HIV status, with subgroup differences tested by the between-subgroup Q (interaction) test, and the influence of duration was examined further by random-effects meta-regression on duration as a continuous covariate. Small-study effects were assessed by funnel-plot inspection, the Egger test, and the trim-and-fill procedure [29, 30]. Robustness was examined by restricting the analysis to trials, by excluding the single high-risk study, and by leave-one-out omission. Analyses were performed in R version 4.4 with the meta and metafor packages [31]. The level of statistical significance was set at *p* < 0.05, and all tests were two-tailed.

## Results

### Study selection

**Fig. 1:**
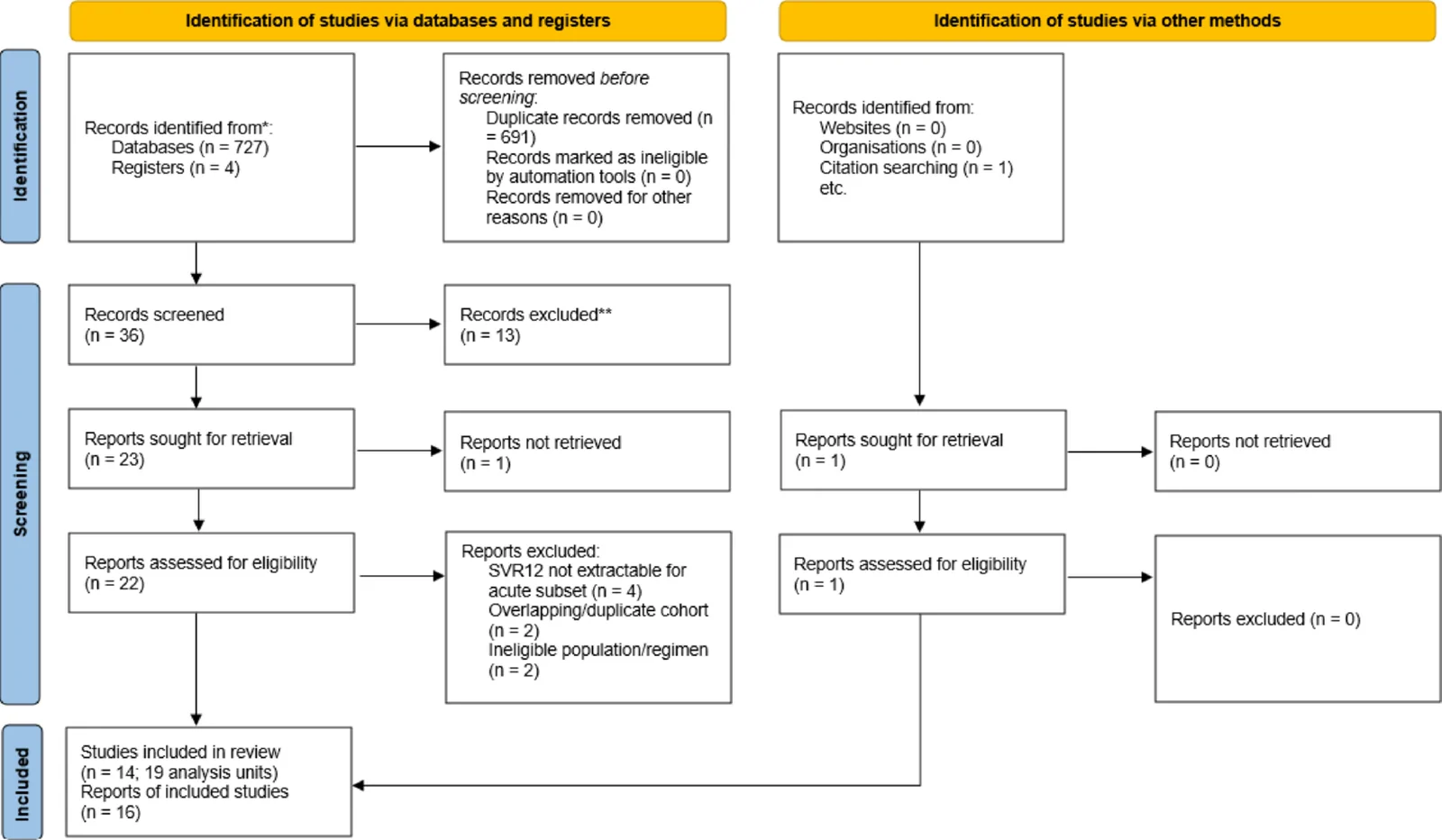
PRISMA 2020 flow diagram of study identification, screening, and inclusion

The database and register searches identified 731 records. Thirty-six records were screened, 13 were excluded at title and abstract, and 23 reports were sought for retrieval; one conference abstract could not be retrieved, leaving 22 reports assessed for eligibility. Eight reports were excluded at full text (four for an SVR12 outcome that could not be isolated for the acute dual-DAA subset, two for overlapping cohorts without independent data, and two for an ineligible population or regimen), and one further report [32] was a companion publication of an included study [33] and was counted only once, yielding 13 studies from the database and register searches. One additional eligible trial [34] was subsequently identified through citation searching of the included studies and relevant reviews, resulting in 14 studies [15–17, 33–43] that contributed 19 single-arm analysis units to the synthesis (Fig. 1).

### Characteristics of included studies

**Table 1:** Characteristics of the 14 included studies (19 single-arm analysis units)

Study (year)	Country	Design	Population/risk group	Age (y)	Male %	Time to treatment/est. infection duration	GT	Regimen	Dur (wk)	*N*	SVR12 mITT/PP *n*/*N* (%)	SVR12 ITT *n*/*N* (%)	RoB
Matthews (REACT) 2021 [15]	Multinational	RCT (duration)	HIV + 69%; MSM 72%; PWID 21%	M 43.8 ± 10.2	96.8	26 wk (17–35)‡	GT1	SOF/VEL	6	93	76/85 (89.4)	76/93 (81.7)	Mod
	Multinational	RCT (duration)	HIV + 69%; MSM 72%; PWID 21%	M 43.8 ± 10.2	96.8	26 wk (17–35)‡	GT1	SOF/VEL	12	95	86/88 (97.7)	86/95 (90.5)	Mod
Deterding (HepNet IV) 2017 [39]	Germany	Single-arm trial	HIV−; sexual/other	Md 49 (36–54)	60	33 d dx→tx	GT1	LDV/SOF	6	20	20/20 (100.0)	20/20 (100.0)	Low
Martinello 2020 [40]	AU/UK/NZ	Single-arm trial	HIV + 77%; MSM 90%	Md 43 (29–73)	100	29 wk (13–52)‡	GT1	GLE/PIB	6	30	27/28 (96.4)	27/30 (90.0)	Mod
Rockstroh 2017 [34]	Germany/UK	Single-arm trial	HIV + 100%; MSM	Md 43 (25–58)	100	< 24 wk (eligibility)‡	GT1a/4	LDV/SOF	6	26	20/23 (87.0)	20/26 (76.9)	Mod
Martinello (TARGET3D) 2023 [16]	AU/UK/NZ	Single-arm trial	HIV + 70%; GBMSM 91%	Md 46 (20–62)	96	17 wk (11–29)‡	GT1a	GLE/PIB	4	23	18/22 (81.8)	18/23 (78.3)	Mod
Kim (PURGE-C) 2026 [17]	USA/Brazil	Single-arm trial	HIV + 51%; MSM	Md 36 (29–43)	98	31 d dx→enrol	GT1	GLE/PIB	4	45	38/44 (86.4)	38/45 (84.4)	Low
Maasoumy (HepNet-V) 2022 [41]	Germany	Single-arm trial	HIV−; PrEP 65%	M 37.4 ± 9.3	95	43 d dx→tx	GT1a	SOF/VEL	8	20	18/18 (100.0)	18/20 (90.0)	Mod
Boerekamps (DAHHS2) 2019 [42]	NL/Belgium	Single-arm trial	HIV + 91%; MSM 100%	M 47 ± 10	100	4.4 mo‡ (RNA→tx 2.0 mo)	GT1a/4	GZR/EBR	8	80	79/80 (98.8)	75/80 (93.8)	Low
Naggie 2019 [35]	USA	Single-arm trial	HIV + 100%; MSM	Md 46 (38–50)	100	116 d (98–156)	GT1	LDV/SOF	8	27	27/27 (100.0)	27/27 (100.0)	Mod
Llibre 2026 [33]	Multinational	Single-arm trial	HIV + 50%; MSM 79%	Md 43 (35–52)	89.2	85 d (69–135)‡; <6 mo 92.5%	GT1	GLE/PIB	8	286	275/275 (100.0)	275/286 (96.2)	Low
Pol 2025 [43]	Multinational	Retrospective cohort	HIV + 51%; PWID 43%	Md 38 (32–47)	85.1	99.5 d (64–137)‡	GT1	GLE/PIB	8	202	150/151 (99.3)	150/202 (74.3)	Mod
Chromy 2019 [36]	Austria	Observational cohort	HIV + 100%; MSM 92%	Md 42†	95†	118 d (IQR 88)†	GT1a	GLE/PIB	8	11	11/11 (100.0)	11/11 (100.0)	Mod
	Austria	Observational cohort	HIV + 100%; MSM 92%	Md 42†	95†	118 d (IQR 88)†	GT1a	Mixed dual	12	11	11/11 (100.0)	11/11 (100.0)	Mod
Huang 2021 [37]	Taiwan	Retrospective cohort	HIV + 100%; MSM 92%	M 34.5 ± 7.6†	100†	NR	Mixed	SOF/VEL	12	84	82/84 (97.6)	82/84 (97.6)	High
Martin-Carbonero 2024 [38]	Spain	Ambispective cohort	HIV + 100%; MSM	Md 40 (34–46)	100	1.8 mo (0.7–4.6) dx→tx†	GT1a	GZR/EBR	12	10	10/10 (100.0)	10/10 (100.0)	Mod
	Spain	Ambispective cohort	HIV + 100%; MSM	Md 40 (34–46)	100	1.8 mo (0.7–4.6) dx→tx†	GT1a	LDV/SOF	12	29	29/29 (100.0)	29/29 (100.0)	Mod
	Spain	Ambispective cohort	HIV + 100%; MSM	Md 40 (34–46)	100	1.8 mo (0.7–4.6) dx→tx†	GT1a	GLE/PIB	8	46	42/44 (95.5)	42/46 (91.3)	Mod
	Spain	Ambispective cohort	HIV + 100%; MSM	Md 40 (34–46)	100	1.8 mo (0.7–4.6) dx→tx†	GT1a	SOF/VEL	12	31	24/28 (85.7)	24/31 (77.4)	Mod

Age: M, mean ± SD; Md, median (IQR or range), years; age and Male % are study-level. Time to treatment gives each study’s own metric: ‡ estimated total infection duration before treatment; otherwise time from diagnosis or first laboratory evidence of acute HCV to treatment initiation (dx→tx); d, days; wk, weeks; mo, months; NR, not reported. † whole-cohort value where the acute dual-DAA subset was not reported separately (Chromy *N* = 38; Huang *N* = 287; Martin-Carbonero *N* = 133). Population: HIV+, HIV-positive proportion; MSM/GBMSM, men who have sex with men; PWID, people who inject drugs; PrEP, pre-exposure prophylaxis. GT, genotype. Proportions to one decimal; complement to 100% is non-SVR. Huang reported only a treated denominator and no separate ITT, so its treated proportion (82/84) was used for both the ITT and mITT/PP analyses. Regimens: GLE/PIB, glecaprevir/pibrentasvir; SOF/VEL, sofosbuvir/velpatasvir; GZR/EBR, grazoprevir/elbasvir; LDV/SOF, ledipasvir/sofosbuvir. RoB, risk of bias; Mod, moderate

The 14 included studies comprised ten interventional trials and four observational cohorts and enrolled patients across four continents (Table 1). Five dual-DAA regimens were represented, namely glecaprevir/pibrentasvir, sofosbuvir/velpatasvir, grazoprevir/elbasvir, ledipasvir/sofosbuvir, and a mixed dual-DAA category, across four treatment durations of 4, 6, 8, and 12 weeks. Participants were predominantly male (60–100% across studies) with median or mean ages ranging from the mid-30s to the high-40s, and the estimated duration of infection before treatment was short (study medians of approximately one to seven months). HIV coinfection status was mixed across the evidence base, and genotype 1 (predominantly subtype 1a) predominated. The primary mITT/PP analysis comprised 1043 SVR12 events among 1078 treated patients, and the ITT analysis comprised 1039 events among 1169 patients.

### Risk of bias

Under the single-arm case-series appraisal, four studies were at low risk of bias [17, 33, 36, 37], nine at moderate risk, and one at high risk of bias [41]; the high-risk rating reflected an incompletely reconcilable SVR12 denominator within a retrospective care-cascade design. The item-level appraisal and the supplementary Risk of Bias 2 assessment for the single randomised trial are reported in the Supplementary material (Table S1).

### Pooled SVR12

**Fig. 2:**
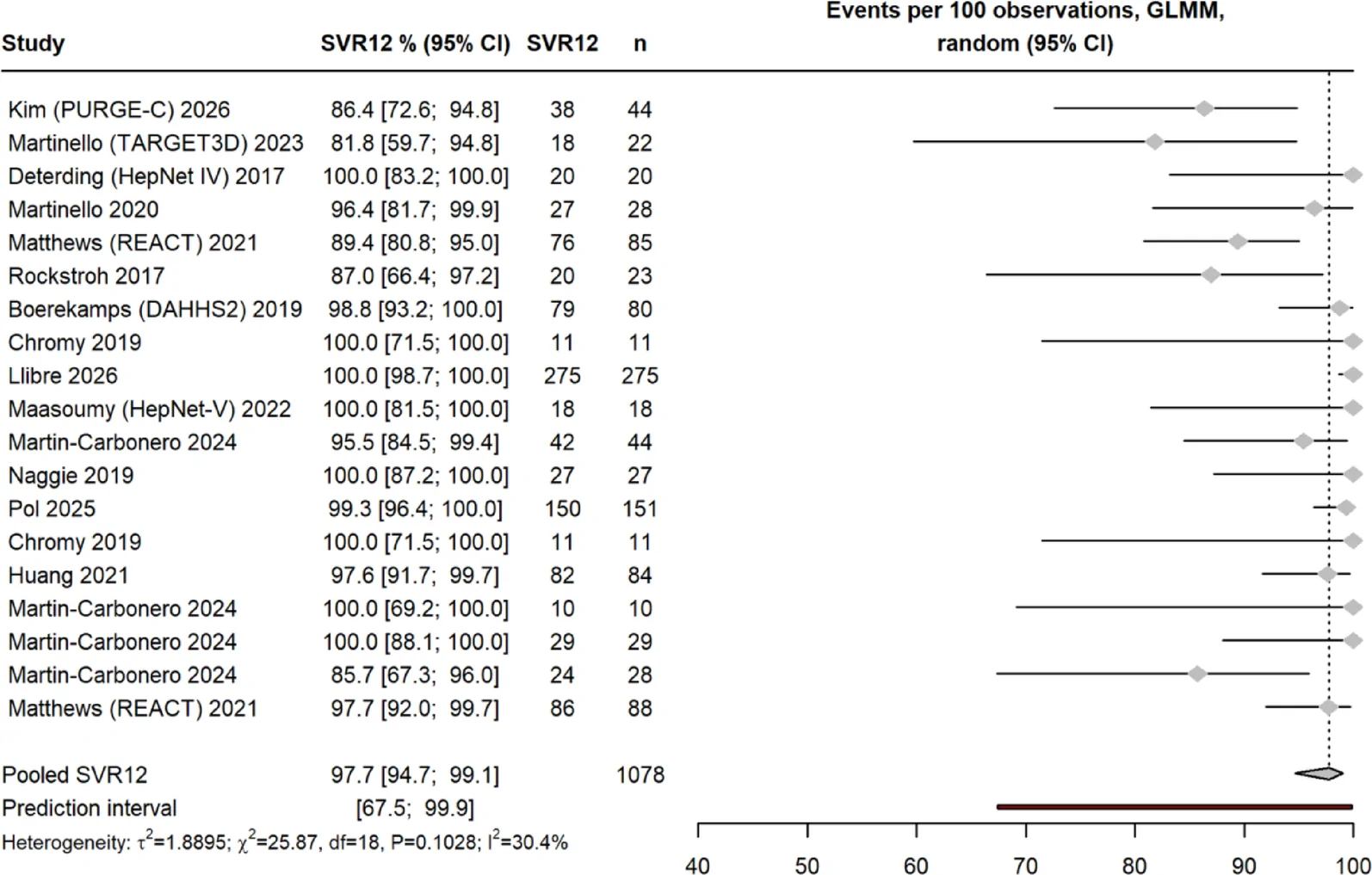
Forest plot of SVR12 in the primary (mITT/PP) analysis

Random-intercept logistic regression model; study-specific proportions with exact 95% Clopper–Pearson confidence intervals and the pooled random-effects estimate with its 95% confidence interval, expressed as percentages. k = 19 analysis units.

In the primary mITT/PP analysis of 19 analysis units (1043 of 1078 patients), the pooled SVR12 was 97.7% (95% CI 94.7–99.1), with a 95% prediction interval of 67.5–99.9% and moderate heterogeneity (I² = 30.4%, 95% CI 0.0–60.1; τ² = 1.89) (Fig. 2; Table 2). Individual-study SVR12 ranged from 81.8% to 100%, and 9 of the 19 analysis units reached 100%; the largest single contributor, Llibre 2026, achieved 275 of 275 (100%). In the pre-specified ITT sensitivity analysis (1039 of 1169 patients), which counts loss to follow-up, death, and reinfection as failures, the pooled SVR12 fell to 92.2% (95% CI 87.3–95.3), with a wider prediction interval (62.6–98.8%) and substantial heterogeneity (I² = 70.8%; τ² = 0.79) (Table 2; Supplementary Figure S1).

**Table 2:** Pooled SVR12 estimates

Analysis	k	SVR12% (95% CI)	95% PI	I²	τ²
SVR12 mITT/PP (primary)	19	97.7 (94.7–99.1)	67.5–99.9	30.4%	1.89
SVR12 ITT (sensitivity)	19	92.2 (87.3–95.3)	62.6–98.8	70.8%	0.79

PI, prediction interval; k, number of analysis units. Estimates from a random-intercept logistic regression model

### Subgroup analyses and meta-regression

**Table 3:** Pre-specified subgroup analyses of SVR12 (primary mITT/PP data)

Moderator	Subgroup	k	SVR12% (95% CI)	I²	*p* (between)
Regimen	GLE/PIB	7	97.8 (90.4–99.5)	51.8%	0.608
	GZR/EBR	2	98.9 (92.5–99.8)	0%	
	LDV/SOF	4	99.5 (50.5–100.0)	0%	
	SOF/VEL	5	95.2 (88.9–98.0)	54.4%	
	Mixed dual-DAA	1	100.0 (single study)	—	
Duration	4 weeks	2	84.8 (74.1–91.6)	0%	< 0.001
	6 weeks	4	91.7 (86.2–95.1)	0%	
	8 weeks	7	99.4 (97.5–99.9)	0%	
	12 weeks	6	97.4 (91.7–99.2)	27.7%	
Design	Trial	11	97.4 (92.0–99.2)	23.4%	0.764
	Cohort	8	98.0 (93.6–99.4)	29.8%	
HIV status	HIV-positive	9	96.8 (90.9–98.9)	0%	0.975
	HIV-negative	2	100.0 (NE)	0%	
	Mixed	8	97.4 (91.5–99.2)	64.6%	

p (between) is the between-subgroup Q (interaction) test, shown once per moderator. NE, not estimable (boundary estimate at 100%)

**Fig. 3:**
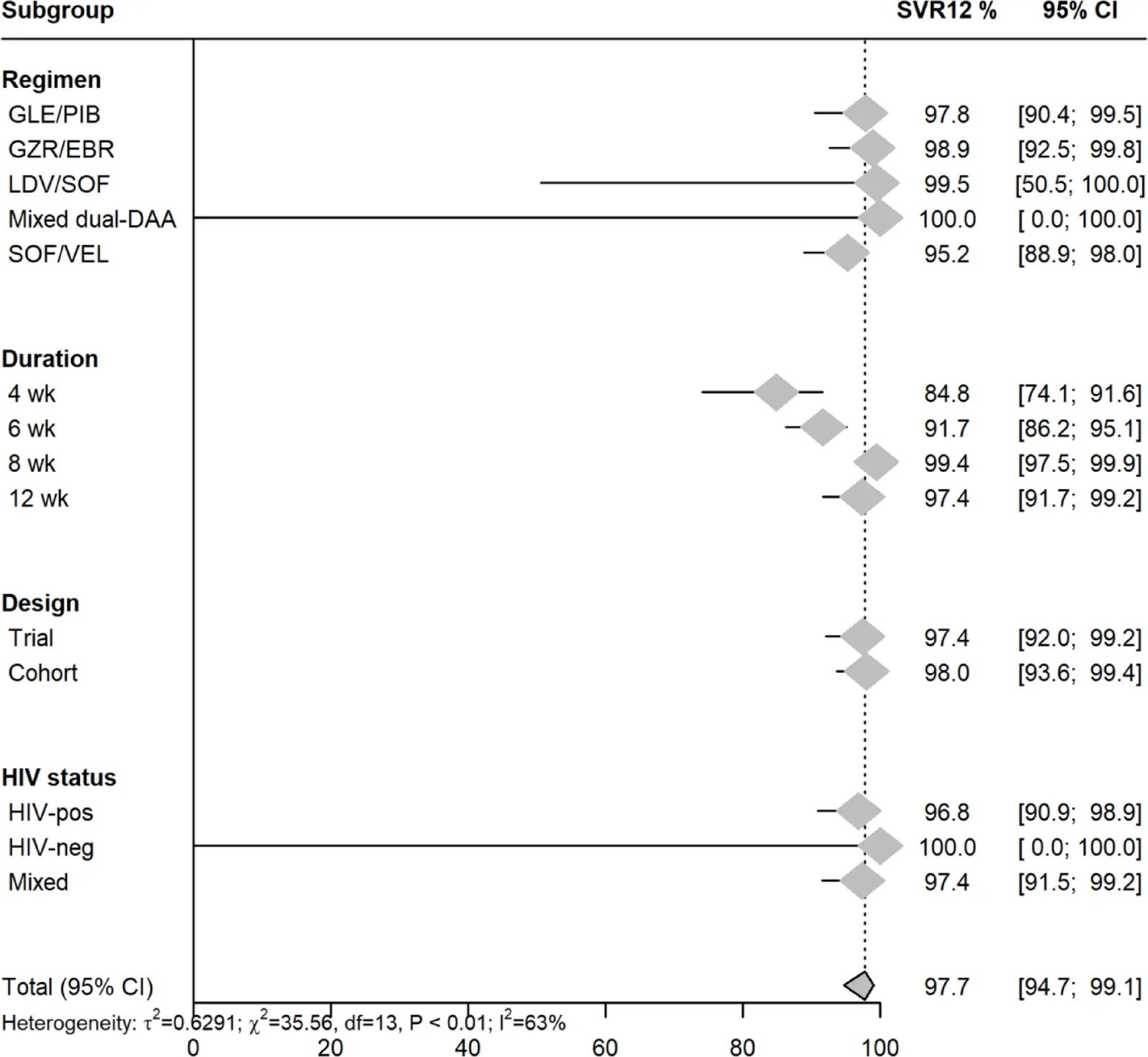
Subgroup analyses of SVR12 by regimen, treatment duration, study design, and HIV status

SVR12 differed significantly across treatment-duration strata (*p* < 0.001): the pooled estimate was 84.8% (95% CI 74.1–91.6) for 4-week regimens, 91.7% (86.2–95.1) for 6 weeks, 99.4% (97.5–99.9) for 8 weeks, and 97.4% (91.7–99.2) for 12 weeks (Table 3). No significant subgroup differences were observed by regimen (*p* = 0.608; glecaprevir/pibrentasvir 97.8%, sofosbuvir/velpatasvir 95.2%, grazoprevir/elbasvir 98.9%, ledipasvir/sofosbuvir 99.5%), by study design (trials 97.4% vs. cohorts 98.0%; *p* = 0.764), or by HIV status (HIV-positive 96.8%, HIV-negative 100.0%, HIV-mixed 97.4%; *p* = 0.975) (Table 3; Fig. 3). On random-effects meta-regression, treatment duration modelled as a continuous covariate was not a significant predictor (β = 0.22 logit units per week; 95% CI − 0.04 to 0.49; *p* = 0.095).

Pooled random-effects SVR12 (%) with 95% confidence intervals for each level of the four pre-specified moderators; between-subgroup heterogeneity tested by the Q (interaction) test. Primary mITT/PP data.

**Fig. 4:**
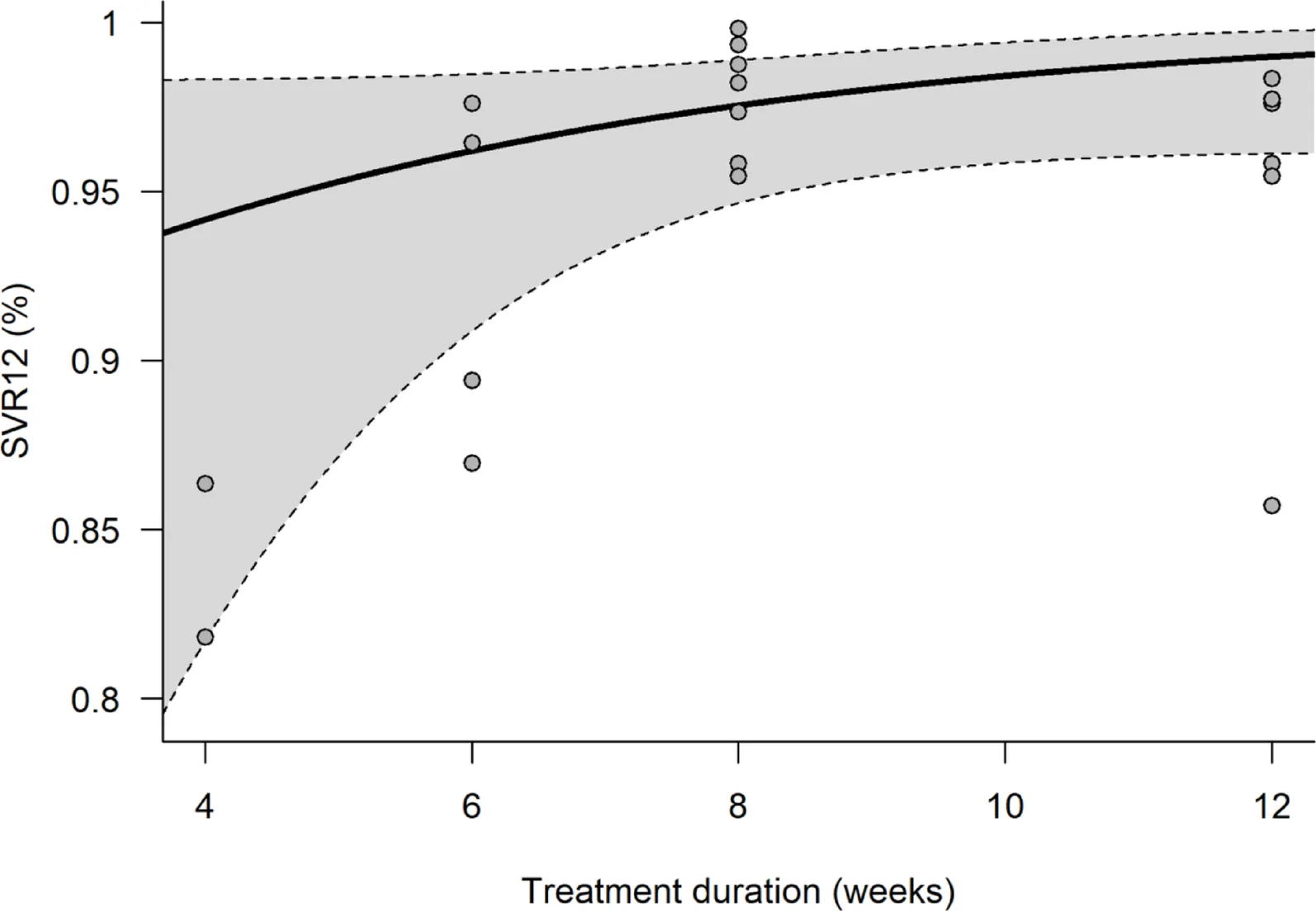
Meta-regression of SVR12 on treatment duration

The between-subgroup interaction was significant only for treatment duration, with the lowest SVR12 in the 4-week stratum and the highest in the 8-week stratum (Fig. 3; Table 3). In random-effects meta-regression on duration as a continuous covariate, the association was not statistically significant (β = 0.22 logit units per additional week, 95% CI − 0.04 to 0.49; *p* = 0.095, Fig. 4).

Bubble plot of study-specific SVR12 (%) against treatment duration (weeks); bubble area is proportional to study precision, and the line is the fitted random-effects meta-regression with its 95% confidence band.

### Sensitivity analyses and small-study effects

The pooled SVR12 was stable across pre-specified sensitivity analyses: 97.4% (95% CI 92.0–99.2) when restricted to trials, 97.8% (94.4–99.2) after excluding the single high-risk study, and 96.8–98.1% across leave-one-out omissions, and the ITT re-analysis gave 92.2% (87.3–95.3). Funnel-plot asymmetry was statistically significant on the Egger test (*p* < 0.001), and trim-and-fill imputation added 9 hypothetical studies and lowered the adjusted estimate to 90.0%; these small-study methods are, however, unreliable for proportions near the ceiling and are reported for completeness rather than as evidence of reporting bias. Full sensitivity and small-study results are provided in the Supplementary material (Tables S2–S4; Figures S1–S3).

## Discussion

In this systematic review and single-arm proportion meta-analysis we estimated the sustained virological response achieved with interferon-free dual direct-acting antiviral therapy in acute and recently acquired hepatitis C. Across 14 studies and 19 analysis units the pooled cure rate remained very high when a completed course was analysed, and it was lower under the conservative intention-to-treat definition. To our knowledge this is the first synthesis to combine the recent short-course dual-DAA studies with the standard-duration evidence, extending an earlier meta-analysis that was confined largely to standard durations and preceded much of the four to eight week glecaprevir and pibrentasvir programme [20]. These findings should be read as pooled estimates from a predominantly single-arm evidence base rather than as comparative efficacy, and they inform the interpretations that follow [15].

The near universal cure observed once a course was completed is consistent with the pangenotypic efficacy of these regimens in chronic infection, where response rates above 95% are routine [3–5]. We found no significant difference between regimens, which suggests that the choice among glecaprevir and pibrentasvir, sofosbuvir and velpatasvir, grazoprevir and elbasvir, and ledipasvir and sofosbuvir can be guided by availability, cost, and drug interactions rather than by expected cure. One plausible contributor to the uniformly high response is the biology of early infection, in which lower viral diversity may render the virus more susceptible to a completed course [9].

The clearest signal in our data was the effect of treatment duration. Pooled cure was lowest for four week regimens and highest for eight-week regimens, and the between-duration difference was significant. This pattern aligns with the randomised REACT trial, in which six weeks of sofosbuvir and velpatasvir was inferior to twelve weeks, and with the four week TARGET3D and PURGE-C studies, which reported the lowest responses in the evidence base [15–17]. The continuous meta-regression was not significant, which reflects the non-monotonic shape of the relationship rather than an absence of association, because cure rose from four to eight weeks and then plateaued. Taken together these results suggest that eight weeks may represent a reasonable balance between brevity and reliability, whereas the shortest four-week courses carry a meaningful risk of failure.

The gap between the completed-course estimate and the intention-to-treat estimate is itself informative. Much of the difference reflects loss to follow-up, non-adherence, and reinfection rather than pharmacological failure, outcomes that are common in the key populations from which these studies drew [18]. Reinfection after cure is well documented among men who have sex with men and people who inject drugs, and late relapse is rare once cure is achieved, so the residual failures under intention-to-treat point to programmatic rather than virological gaps [18, 19]. This distinction matters for policy, because rapid and reliable cure of the index infection supports treatment as prevention and micro-elimination in these networks, an approach associated with declining incidence where access is unrestricted [12, 13].

We detected statistically significant funnel-plot asymmetry, and trim-and-fill imputation lowered the adjusted estimate. We interpret this cautiously, because the relationship between effect size and standard error is mechanically induced when most studies report proportions close to one, so conventional small-study tests are unreliable in this setting and should not be read as strong evidence of reporting bias [24].

Several practical directions follow from these observations. Clinicians may reasonably treat acute and recently acquired hepatitis C early rather than waiting for spontaneous clearance, and current guidance already supports this approach [4, 14]. Where a shortened course is considered, an eight-week regimen appears preferable to a four-week course on present evidence. Future randomised trials could compare four and eight weeks directly within the same population and stratify by baseline viral load, and cohort programmes might strengthen reinfection surveillance and retention so that the high biological cure translates into durable population benefit.

This review has several limitations. The evidence base is predominantly single arm, most studies were at moderate risk of bias, and one retrospective cohort was at high risk, so the pooled estimate carries the confounding and selection concerns inherent to uncontrolled data, which we acknowledged by appraising every study as a case series and by grading the certainty of evidence as low. Definitions of acute and recently acquired infection and of the analysis population varied across studies, and treatment duration and regimen were partly confounded because the four-week arms were all glecaprevir and pibrentasvir, which limits separation of regimen and duration effects. The four- and six-week strata contained few studies, the population was weighted towards men who have sex with men living with HIV, which may limit generalisability to other groups, and funnel-based methods were of limited value for proportions near the ceiling. We mitigated these issues through a full re-verification of every extracted proportion, pre-specified sensitivity analyses, and leave-one-out testing, all of which left the estimate essentially unchanged.

Interferon-free dual direct-acting antiviral therapy cures almost all people with acute or recently acquired hepatitis C, with eight-week regimens performing best and four week courses least well. Given the largely single-arm evidence, randomised comparisons of treatment duration are needed to confirm these findings.

## Supplementary Information

Supplementary Material 1

Supplementary Material 2

## Data Availability

The extracted dataset and analysis code are available from the corresponding author on reasonable request.

## Declarations

### Registration

The review protocol was prepared a priori and submitted to the International Prospective Register of Systematic Reviews (PROSPERO). The registration number is CRD420261464486.
