# Supplementary material for: Efficacy of direct-acting antiviral regimens for acute HCV infection: a systematic review and meta-analysis
Source: Virol J. 2026 Oct 7;23:240. doi: 10.1186/s12985-026-03307-x (PMC13647546; doi:10.1186/s12985-026-03307-x)
Supplement: Supplementary file 1 [file 12985_2026_3307_MOESM1_ESM.docx]

**PRISMA 2020 Checklist**

*Efficacy of direct-acting antiviral regimens for acute HCV infection: a systematic review and meta-analysis*

*Locations refer to sections of the main manuscript (M) and Supplementary material (S). From: Page MJ, et al. The PRISMA 2020 statement: an updated guideline for reporting systematic reviews. BMJ 2021;372:n71.*

| **Section / Topic** | **#** | **Checklist item** | **Location where item is reported** |
| --- | --- | --- | --- |
| **TITLE** | | | |
| Title | 1 | Identify the report as a systematic review. | M: Title page; Manuscript title |
| **ABSTRACT** | | | |
| Abstract | 2 | See the PRISMA 2020 for Abstracts checklist. | M: Abstract (structured: Background, Methods, Results, Conclusion) |
| **INTRODUCTION** | | | |
| Rationale | 3 | Describe the rationale for the review in the context of existing knowledge. | M: 1. Introduction |
| Objectives | 4 | Provide an explicit statement of the objective(s) or question(s) the review addresses. | M: 1. Introduction (final paragraph) |
| **METHODS** | | | |
| Eligibility criteria | 5 | Specify the inclusion and exclusion criteria for the review and how studies were grouped for the syntheses. | M: 2.2 Eligibility criteria |
| Information sources | 6 | Specify all databases, registers, websites, organisations, reference lists and other sources searched or consulted; specify the date each was last searched. | M: 2.3 Information sources and search strategy; S: S1.1 |
| Search strategy | 7 | Present the full search strategies for all databases, registers and websites, including any filters and limits used. | M: 2.3; S: S1.1 Full electronic search strategy |
| Selection process | 8 | Specify the methods used to decide whether a study met the inclusion criteria, including how many reviewers screened each record and each report, whether they worked independently, and if applicable, details of automation tools used. | M: 2.4 Study selection and data extraction |
| Data collection process | 9 | Specify the methods used to collect data from reports, including how many reviewers collected data from each report, whether they worked independently, any processes for obtaining or confirming data from investigators, and if applicable, automation tools used. | M: 2.4 Study selection and data extraction |
| Data items | 10a | List and define all outcomes for which data were sought. Specify whether all results compatible with each outcome domain were sought, and if not, the methods used to decide which results to collect. | M: 2.4; 2.6 (primary outcome SVR12) |
| Data items | 10b | List and define all other variables for which data were sought (e.g. participant and intervention characteristics, funding sources). Describe any assumptions made about missing or unclear information. | M: 2.4; Table 1 |
| Study risk of bias assessment | 11 | Specify the methods used to assess risk of bias in the included studies, including details of the tool(s) used, how many reviewers assessed each study and whether they worked independently, and if applicable, automation tools used. | M: 2.5 Risk-of-bias assessment (JBI case-series tool); S: S2 |
| Effect measures | 12 | Specify for each outcome the effect measure(s) (e.g. risk ratio, mean difference) used in the synthesis or presentation of results. | M: 2.6 Statistical analysis (proportion / SVR12) |
| Synthesis methods | 13a | Describe the processes used to decide which studies were eligible for each synthesis (e.g. tabulating the study intervention characteristics and comparing against the planned groups for each synthesis). | M: 2.6; S: S1.2 Synthesis model and rationale |
| Synthesis methods | 13b | Describe any methods required to prepare the data for presentation or synthesis, such as handling of missing summary statistics, or data conversions. | M: 2.6; S: S1.2 |
| Synthesis methods | 13c | Describe any methods used to tabulate or visually display results of individual studies and syntheses. | M: 2.6 (forest plots, tables); Figures 2–4; Tables 2–3 |
| Synthesis methods | 13d | Describe any methods used to synthesise results and provide a rationale for the choice(s). If meta-analysis was performed, describe the model(s), method(s) to identify the presence and extent of statistical heterogeneity, and software package(s) used. | M: 2.6 (random-intercept logistic regression; τ²/ML; I²; meta/metafor R packages); S: S1.2 |
| Synthesis methods | 13e | Describe any methods used to explore possible causes of heterogeneity among study results (e.g. subgroup analysis, meta-regression). | M: 2.6 (pre-specified subgroups and meta-regression); Results 3.5 |
| Synthesis methods | 13f | Describe any sensitivity analyses conducted to assess robustness of the synthesised results. | M: 2.6; Results 3.6; S: S3, Tables S2–S3 |
| Reporting bias assessment | 14 | Describe any methods used to assess risk of bias due to missing results in a synthesis (arising from reporting biases). | M: 2.6; S: S1.4 (Egger test, trim-and-fill); Table S4 |
| Certainty assessment | 15 | Describe any methods used to assess certainty (or confidence) in the body of evidence for an outcome. | M: 2.6; S: S1.4 Certainty of evidence |
| **RESULTS** | | | |
| Study selection | 16a | Describe the results of the search and selection process, from the number of records identified in the search to the number of studies included in the review, ideally using a flow diagram. | M: 3.1 Study selection; Figure 1 (PRISMA flow) |
| Study selection | 16b | Cite studies that might appear to meet the inclusion criteria, but which were excluded, and explain why they were excluded. | M: 3.1; Figure 1 (exclusions with reasons) |
| Study characteristics | 17 | Cite each included study and present its characteristics. | M: 3.2 Characteristics of included studies; Table 1 |
| Risk of bias in studies | 18 | Present assessments of risk of bias for each included study. | M: 3.3 Risk of bias; S: Table S1 |
| Results of individual studies | 19 | For all outcomes, present, for each study, summary statistics for each group (where appropriate) and an effect estimate and its precision (e.g. confidence/credible interval), ideally using structured tables or plots. | M: 3.4; Figure 2 (forest plot); Table 2 |
| Results of syntheses | 20a | For each synthesis, briefly summarise the characteristics and risk of bias among contributing studies. | M: 3.2–3.4; Tables 1–2 |
| Results of syntheses | 20b | Present results of all statistical syntheses conducted. If meta-analysis was done, present for each the summary estimate and its precision (e.g. confidence/credible interval) and measures of statistical heterogeneity. If comparing groups, describe the direction of the effect. | M: 3.4 Pooled SVR12 (97.7%, 95% CI 94.7–99.1; I²=30.4%); Figure 2; Table 2 |
| Results of syntheses | 20c | Present results of all investigations of possible causes of heterogeneity among study results. | M: 3.5 Subgroup analyses and meta-regression; Figures 3–4; Table 3 |
| Results of syntheses | 20d | Present results of all sensitivity analyses conducted to assess the robustness of the synthesised results. | M: 3.6 Sensitivity analyses; S: Tables S2–S3, Figures S1, S3 |
| Reporting biases | 21 | Present assessments of risk of bias due to missing results (arising from reporting biases) for each synthesis assessed. | M: 3.6 small-study effects; S: Table S4, Figure S2 (funnel) |
| Certainty of evidence | 22 | Present assessments of certainty (or confidence) in the body of evidence for each outcome assessed. | M: 3.6 / 4. Discussion; S: S1.4 |
| **DISCUSSION** | | | |
| Discussion | 23a | Provide a general interpretation of the results in the context of other evidence. | M: 4. Discussion |
| Discussion | 23b | Discuss any limitations of the evidence included in the review. | M: 4. Discussion (limitations paragraph) |
| Discussion | 23c | Discuss any limitations of the review processes used. | M: 4. Discussion (limitations paragraph) |
| Discussion | 23d | Discuss implications of the results for practice, policy, and future research. | M: 4. Discussion (final paragraphs) |
| **OTHER INFORMATION** | | | |
| Registration and protocol | 24a | Provide registration information for the review, including the register name and registration number, or state that the review was not registered. | M: 2.1; Declarations: Registration (PROSPERO) |
| Registration and protocol | 24b | Indicate where the review protocol can be accessed, or state that a protocol was not prepared. | M: 2.1; Declarations: Registration |
| Registration and protocol | 24c | Describe and explain any amendments to information provided at registration or in the protocol. | M: 2.1; S: S1.3 Deviation from the registered primary analysis |
| Support | 25 | Describe sources of financial or non-financial support for the review, and the role of the funders or sponsors in the review. | M: Declarations: Funding |
| Competing interests | 26 | Declare any competing interests of review authors. | M: Declarations: Competing interests |
| Availability of data, code and other materials | 27 | Report which of the following are publicly available and where they can be found: template data collection forms; data extracted from included studies; data used for all analyses; analytic code; any other materials used in the review. | M: Declarations: Availability of data and materials; S: S5 Full analysis dataset; AcuteHCV_DualDAA_Master_Final.xlsx |
