# Supplementary material for: Efficacy of direct-acting antiviral regimens for acute HCV infection: a systematic review and meta-analysis
Source: Virol J. 2026 Oct 7;23:240. doi: 10.1186/s12985-026-03307-x (PMC13647546; doi:10.1186/s12985-026-03307-x)
Supplement: Supplementary file 2 [file 12985_2026_3307_MOESM2_ESM.docx]

**Supplementary material**

# S1. Supplementary Methods

## S1.1 Full electronic search strategy

The MEDLINE (PubMed) strategy combined three concept blocks with the Boolean operator AND, without a design filter. Block 1 (hepatitis C): "hepatitis C"[MeSH] OR "hepatitis C"[tiab] OR HCV[tiab] OR hepacivirus[tiab]. Block 2 (acute / recently acquired): acute[tiab] OR "recently acquired"[tiab] OR "recent infection"[tiab] OR "recent HCV"[tiab] OR "early infection"[tiab] OR "early hepatitis C"[tiab] OR seroconversion[tiab] OR incident[tiab]. Block 3 (direct-acting antivirals): "antiviral agents"[MeSH] OR "direct-acting antiviral*"[tiab] OR DAA[tiab] OR DAAs[tiab] OR sofosbuvir[tiab] OR velpatasvir[tiab] OR ledipasvir[tiab] OR glecaprevir[tiab] OR pibrentasvir[tiab] OR grazoprevir[tiab] OR elbasvir[tiab] OR daclatasvir[tiab] OR ombitasvir[tiab] OR paritaprevir[tiab]. Equivalent Scopus TITLE-ABS-KEY and Cochrane CENTRAL strings, together with the register searches (ClinicalTrials.gov, WHO ICTRP), are recorded in the accompanying search log. Searches were run on 1 July 2026 and covered 1 January 2011 onward.

## S1.2 Synthesis model and rationale

Single proportions were pooled with a random-intercept logistic regression (generalised linear mixed) model, which models the binomial numerator and denominator of each study directly on the logit scale and avoids the instability of variance-stabilising transformations when observed proportions approach 1. This model was preferred to the Freeman–Tukey double-arcsine transformation specified in the registered protocol, because the double-arcsine method can yield non-interpretable back-transformed intervals and biased pooled estimates when study sizes are heterogeneous and proportions are near the ceiling, as is the case for SVR12 in the DAA era. Between-study heterogeneity (τ²) was estimated by maximum likelihood, individual-study intervals were computed by the exact Clopper–Pearson method, and the 95% prediction interval was based on a t-distribution with k − 1 degrees of freedom. Heterogeneity was summarised by I², τ², and the Q test; for the primary analysis I² was 30.4% (95% CI 0.0–60.1) with τ² = 1.89.

## S1.3 Deviation from the registered primary analysis

The registered protocol nominated the ITT proportion as the primary effect measure. During analysis, the mITT/PP proportion was adopted as primary because it estimates the virological efficacy of a completed dual-DAA course, whereas the ITT proportion — which counts loss to follow-up, death, and reinfection as failures — is a conservative composite that mixes efficacy with programmatic and behavioural outcomes. Both measures are reported in full, and the ITT proportion is presented as a pre-specified sensitivity analysis, so that the change in primary measure does not selectively favour a more optimistic estimate.

## S1.4 Small-study effects and certainty of evidence

Small-study effects were assessed by funnel-plot inspection, the Egger regression test, and the Duval and Tweedie trim-and-fill procedure. These methods are of limited validity for single-proportion meta-analyses in which most studies report proportions close to 100%, because the relationship between effect size and its standard error is then mechanically induced rather than indicative of reporting bias; the results in Section S4 should be interpreted with this caveat. The certainty of the pooled evidence was appraised with the GRADE approach, which begins at low certainty for single-arm (non-comparative) proportions and may be further downgraded for risk of bias, inconsistency, indirectness, imprecision, or publication bias.

# S2. Risk of bias (detailed)

| **Study** | **I1** | **I2** | **I3** | **I4** | **I5** | **I6** | **I7** | **I8** | **I9** | **Overall** |
| --- | --- | --- | --- | --- | --- | --- | --- | --- | --- | --- |
| Matthews (REACT) 2021 | Y | Y | Y | U | Y | Y | Y | Y | Y | Mod |
| Deterding (HepNet IV) 2017 | Y | Y | Y | U | Y | Y | Y | Y | U | Low |
| Martinello 2020 | Y | Y | Y | U | Y | Y | Y | Y | Y | Mod |
| Rockstroh 2017 | Y | Y | Y | U | Y | Y | Y | U | Y | Mod |
| Martinello (TARGET3D) 2023 | Y | Y | Y | U | U | Y | Y | Y | Y | Mod |
| Kim (PURGE-C) 2026 | Y | Y | Y | U | Y | Y | Y | Y | Y | Low |
| Maasoumy (HepNet-V) 2022 | Y | Y | Y | U | Y | Y | Y | Y | U | Mod |
| Boerekamps (DAHHS2) 2019 | Y | Y | Y | U | Y | Y | Y | Y | Y | Low |
| Naggie 2019 | Y | Y | Y | U | Y | Y | Y | Y | Y | Mod |
| Llibre 2026 | Y | Y | Y | U | Y | Y | Y | Y | Y | Low |
| Pol 2025 | Y | U | Y | Y | U | Y | Y | N | U | Mod |
| Chromy 2019 | Y | Y | Y | Y | U | Y | Y | Y | Y | Mod |
| Huang 2021 | Y | Y | Y | Y | U | U | U | N | Y | High |
| Martin-Carbonero 2024 | Y | Y | Y | U | U | Y | Y | N | Y | Mod |

*Table S1. Item-level single-arm (JBI Case Series) appraisal. Items: I1 inclusion criteria clear; I2 condition measured in a standard, reliable way; I3 valid identification methods; I4 consecutive inclusion; I5 complete inclusion; I6 demographics reported; I7 clinical information reported; I8 outcomes and follow-up complete; I9 site/clinic demographics reported. Y, yes; N, no; U, unclear. Mod, moderate. Overall counts: 4 low, 9 moderate, 1 high.*

The single randomised controlled trial (REACT) was additionally appraised with the Cochrane Risk of Bias 2 tool and judged to raise some concerns overall, driven principally by the open-label design; because REACT contributed two single-arm duration proportions rather than a randomised contrast to the synthesis, the single-arm appraisal remained the primary basis for its rating.

# S3. Sensitivity analyses

| **Analysis** | **k** | **SVR12 % (95% CI)** | **I²** |
| --- | --- | --- | --- |
| Primary (all units, mITT/PP) | 19 | 97.7 (94.7–99.1) | 30.4% |
| Trials only | 11 | 97.4 (92.0–99.2) | 23.4% |
| Excluding high risk-of-bias study (Huang) | 18 | 97.8 (94.4–99.2) | 24.0% |
| ITT (all units) | 19 | 92.2 (87.3–95.3) | 70.8% |

*Table S2. Pre-specified sensitivity analyses of the pooled SVR12 proportion.*

The pooled SVR12 was robust to restriction to trials and to exclusion of the single high-risk study, and the ITT re-analysis produced a lower but still high pooled estimate with greater heterogeneity (Table S2).

| **Study omitted** | **SVR12 % (95% CI)** | **I²** |
| --- | --- | --- |
| None (pooled) | 97.7 (94.7–99.1) | 30.4% |
| Llibre 2026 | 96.8 (93.5–98.5) | 34.3% |
| Pol 2025 | 97.5 (94.0–99.0) | 10.3% |
| Rockstroh 2017 | 98.0 (95.1–99.2) | 32.3% |
| Boerekamps (DAHHS2) 2019 | 97.7 (94.2–99.1) | 22.6% |
| Kim (PURGE-C) 2026 | 98.1 (95.2–99.2) | 28.7% |
| Huang 2021 | 97.8 (94.4–99.2) | 24.0% |
| Range across all 19 omissions | 96.8–98.1 | 10.3–34.3% |

*Table S3. Leave-one-out sensitivity analysis (primary mITT/PP data). Selected omissions shown; the pooled estimate ranged from 96.8% to 98.1% across all 19 omissions.*

**Figure S3. Leave-one-out sensitivity analysis of pooled SVR12.**

Pooled random-effects SVR12 (%) with 95% confidence interval recomputed after omitting each analysis unit in turn. Primary mITT/PP data.

No single analysis unit materially altered the pooled estimate; the largest reduction followed omission of Llibre 2026 (to 96.8%) (Table S3, Figure S3).

# S4. Intention-to-treat analysis and small-study effects

**Figure S1. Forest plot of SVR12 in the ITT sensitivity analysis.**

Random-intercept logistic regression model; study-specific proportions with exact 95% Clopper–Pearson confidence intervals and the pooled random-effects estimate with its 95% confidence interval. k = 19 analysis units.

In the ITT analysis, the pooled SVR12 was 92.2% (95% CI 87.3–95.3) with substantial heterogeneity (I² = 70.8%; τ² = 0.79) and a prediction interval of 62.6–98.8% (Figure S1).

| **Analysis** | **k** | **Egger p** | **Imputed (k0)** | **Adjusted SVR12 %** |
| --- | --- | --- | --- | --- |
| SVR12 mITT/PP (primary) | 19 | <0.001 | 9 | 90.0 |
| SVR12 ITT (sensitivity) | 19 | 0.007 | 8 | 83.7 |

*Table S4. Small-study-effect assessment. Egger p, p-value of the Egger regression test; k0, number of studies imputed by trim-and-fill; adjusted SVR12, pooled estimate after trim-and-fill imputation. These methods are of limited validity for proportions near 100% (Section S1.4).*

**Figure S2. Funnel plots for the primary (mITT/PP) and ITT analyses.**

Panel A, primary mITT/PP analysis; Panel B, ITT analysis. Logit-transformed SVR12 against its standard error.

Funnel-plot asymmetry was statistically significant on the Egger test for both analyses, and trim-and-fill imputation lowered the adjusted pooled estimate to 90.3% (mITT/PP) and 84.1% (ITT); these values should be interpreted in light of the ceiling-effect caveat described in Section S1.4 (Table S4, Figure S2).

# S5. Full analysis dataset

The complete analysis-ready dataset (19 single-arm analysis units with regimen, treatment duration, design, HIV status, genotype, treatment number, and the verified SVR12 numerators and denominators under both ITT and mITT/PP definitions), together with the variable dictionary, is provided as the accompanying spreadsheet (AcuteHCV_DualDAA_Master_Final.xlsx). Two definitional points are recorded there for transparency: the 12-week Chromy dual-DAA unit (11/11) includes two ritonavir-boosted regimen patients and would become 9/9 if ritonavir-boosted regimens were excluded; and the DAHHS2 ITT and mITT/PP values are harmonised to the review convention (reinfection counted as failure under ITT), which reverses the source paper's own labels while preserving the numerators and denominators.

The completed PRISMA 2020 checklist is provided as a separate file.
